# Miscellany

**Published:** 1905-10

**Authors:** 


### MISCELLANY.

Civil Service Examinations for the State and County Service.—The State Civil Service Commission has announced a general examination to be held October 28, 1905. The positions included in this examination are those of assistant in botany, education department, $600; assistant in microscopy, Buffalo Cancer Laboratory, $720; bertillon clerk, State prisons, $900; woman bookkeeper, 4th grade, $480 to $720; bridge draughtsman, State engineer's office, $1,200; deputy factory inspector, $1,200; orderly, Erie County Hospital, $540 and maintenance; page, State and county offices, $240 to $360; prison guard, State prisons, $660; rodman, State engineer's department, $3.50 per day; teacher, State institutions, $300 to $600 and maintenance. The last day for filing applications is October 23d; application forms and detailed information may be obtained by addressing the chief examiner of the commission at Albany.

Battle and Company, Saint Louis, have issued recently the seventh of a series of twelve illustrations of intestinal parasites, which they will send free to physicians on application.

